# Unmasking the ‘rods and rings’ antinuclear antibody pattern: a sign of hepatitis C treatment and a risk factor for cerebrovascular disease

**DOI:** 10.1080/07853890.2025.2600753

**Published:** 2025-12-18

**Authors:** Ko-Ming Lin, Kuang-Hui Yu, Chao-Yang Hsiao, Yu-Jih Su, Tien-Ming Chan

**Affiliations:** ^a^ Division of Rheumatology, Allergy and Immunology, Department of Internal Medicine, Chiayi Chang Gung Memorial Hospital, Chiayi, Taiwan; ^b^ Division of Rheumatology, Allergy, and Immunology, Department of Internal Medicine, Chang Gung Memorial Hospital, Linkou and Chang Gung University, Taoyuan, Taiwan; ^c^ Division of Rheumatology, Allergy and Immunology, and Geriatrics, Department of Internal Medicine, Kaohsiung Chang Gung Memorial Hospital and Chang Gung University College of Medicine, Kaohsiung, Taiwan

**Keywords:** Antinuclear antibody, cerebrovascular disease, hepatitis C, rods and rings

## Abstract

**Introduction:**

The rods and rings (RR) antinuclear antibody (ANA) pattern (AC-23) is predominantly associated with hepatitis C virus (HCV) treatment, particularly involving ribavirin (RBV). Its relevance in other clinical conditions is unclear. This study aims to explore the RR ANA pattern’s associations with clinical conditions, including potential links to cerebrovascular disease (CVD).

**Objectives:**

To investigate the clinical characteristics of patients displaying the RR ANA pattern, assess its associations with autoimmune diseases and other conditions and evaluate the potential risk of CVD in patients with high ANA titres.

**Materials and methods:**

This retrospective study analyzed 43,633 ANA samples collected at Chang Gung Memorial Hospital, Taiwan (August 2019–June 2021). ANA titres ≥1:160 were positive; the RR ANA pattern was identified using the EUROPattern system. Data were obtained from medical records. Statistical analyses (Mann–Whitney U, chi-squared, Fisher’s exact tests) were conducted, with *p* <.05 considered significant.

**Results:**

Among 43,633 samples, 9,999 (22.9%) were ANA-positive, with 55 samples (0.56%) from 35 patients showing the RR ANA pattern. Most (88.6%) RR-positive patients had HCV infection, with 92.3% receiving RBV treatment. Liver comorbidities (cirrhosis 38.7%, fatty liver 51.6%, hepatocellular carcinoma 25.8%) were prevalent. Among four HCV-negative patients, three were on beta-blockers. Patients with high ANA titres (≥1:640) showed a significantly higher prevalence of CVD than those with low titres (≤1:320, *p* = .026). Inflammatory markers and diseases linked to inosine monophosphate dehydrogenase expression may contribute to the CVD risk.

**Discussion and conclusions:**

This study confirms a strong association between the RR ANA pattern and HCV treatment, while suggesting a potential link to CVD at high ANA titres. RR positivity in HCV-negative patients also suggests other triggers, including beta-blockers. These findings underscore the need for further research into the broader clinical implications of the RR ANA pattern and its association with inflammatory diseases.

**KEY MESSAGE**
1. The anti-rods and rings (anti-RR) ANA pattern, once considered specific to HCV therapy, can also appear in non-HCV patients.
2. High-titre anti-RR positivity may be associated with an increased risk of cerebrovascular disease.

## Introduction

Antinuclear antibody (ANA) as detected by the indirect immunofluorescence assay in HEp-2 cells is used to screen for autoimmune diseases. However, the specificity of the ANA test varies according to the titre [1]. The prevalence of ANA is also higher among females and older individuals [2,3]. From the perspective of screening, ANA detection is extremely practical regardless of any clinical manifestations [4]. Increasing evidence also suggests that ANA positivity may be associated with other conditions, including subclinical atherosclerosis, even in the absence of a defined autoimmune diagnosis [5,6], although the relative risk associated with specific ANA indirect immunofluorescence patterns remains underexplored.

In 2014, the International Consensus on ANA Patterns (ICAP) recommended that ANA can be categorized into at least 29 patterns [7]. Ten years later, in 2024, ANA patterns were increased to 31 types [8]. Among these patterns, AC-23 denotes the ‘rods and rings’ (RR) pattern, which is typified by distinct rod and ring structures in the cytoplasm of interphase cells [9]. The RR pattern is created in response to an antigen against inosine monophosphate dehydrogenase (IMPDH) [10].

IMPDH is a purine biosynthetic enzyme and it is tightly regulated at multiple levels. IMPDH plays a key role in purine biosynthesis by catalysing the nicotinamide adenine dinucleotide (NAD+)-dependent oxidation of inosine phosphate (IMP) to xanthosine monophosphate (XMP). This is the first and most important rate-limiting step in the de novo biosynthesis of guanine nucleotides from IMP [11,12]. IMPDH is therefore critical for processes involved in cell proliferation processes such as DNA and RNA synthesis, signal transduction, energy transfer and glycoprotein synthesis. Furthermore, IMPDH regulates lymphocyte activation and proliferation, influencing immune responses.

This RR pattern was usually considered to be more frequent in the setting of treatment for hepatitis C [10,13–17]. In the treatment of hepatitis C virus (HCV) infection, ribavirin (RBV), which irreversibly inhibits IMPDH, the main target of anti-RR antibody, has been an important agent [10,15]. Therefore, some researchers strongly considered this pattern to be related to HCV infection and treatment with pegylated interferon-alpha (IFN-α) and RBV. However, some researchers suggested that anti-RR antibody might also be related to the immune response induced by hepatitis C infection [14,16]. In addition to being closely related to antiviral therapy, anti-RR antibodies have been reported to be related to drug-induced RR structures in cells, including mycophenolic acid, methotrexate, azathioprine and acyclovir [11,16]. These antibodies were also reported to be associated with some other chronic diseases such as hepatitis B infection, chronic kidney disease, diabetes mellitus, chronic obstructive pulmonary disease and hypertension [18]. A study in Türkiye also found that anti-RR antibodies increased after the COVID-19 pandemic than before the pandemic [19]. In addition, this antibody also appears in a certain proportion of patients with autoimmune diseases [19,20]. As the manifestations of this pattern in other diseases are unknown, we can explore other aspects only through detailed investigation and retrospective analysis. This study aimed to explore the clinical and diagnostic relevance of anti-RR positivity and its association with related diseases, in order to clarify its potential implications for physicians and patients.

## Methods

### Study population

This study received approval from the Institutional Review Board of Chang Gung Memorial Hospital under protocol number 202101542B0C604. This retrospective study analyzed samples collected by the Laboratory Medicine Department of Linkou Chang Gung Memorial Hospital (Taoyuan City, Taiwan) from August 2019 to June 2021. ANA titres ≥ 1:160 were considered positive. We reviewed patients’ charts to gather demographic data and clinical characteristics. Other laboratory data were collected at the time of ANA testing.

### Anti-RR antibody (AC-23) analysis

Serum samples were evaluated by standard immunofluorescence ANA testing using a commercial HEp-2 ANA slide (EUROIMMUN Medizinische Labordiagnostika AG, Lübeck, Germany). The ANA pattern was interpreted by EUROPattern (EUROIMMUN). We collected clinical data when the AC-23 pattern first appeared.

Hepatic sonography was performed by a hepatologist. The diagnosis of liver cirrhosis was made using a scoring system that considered four factors: liver surface, liver parenchyma, hepatic vessel and spleen size [21]. Nonalcoholic fatty liver disease was diagnosed according to the findings of hepatorenal echo contrast, liver brightness, deep attenuation and vessel blurring [22].

### Statistical analysis

Statistical evaluation was performed using the Statistical Package for the Social Sciences version 11 (IBM, Armonk, NY, USA). *p* < .05 was considered significant.

The nonparametric Mann–Whitney *U* test, chi-squared test and Fisher’s exact test were performed for comparisons between the groups.

## Results

### Study population

**Figure 1.**
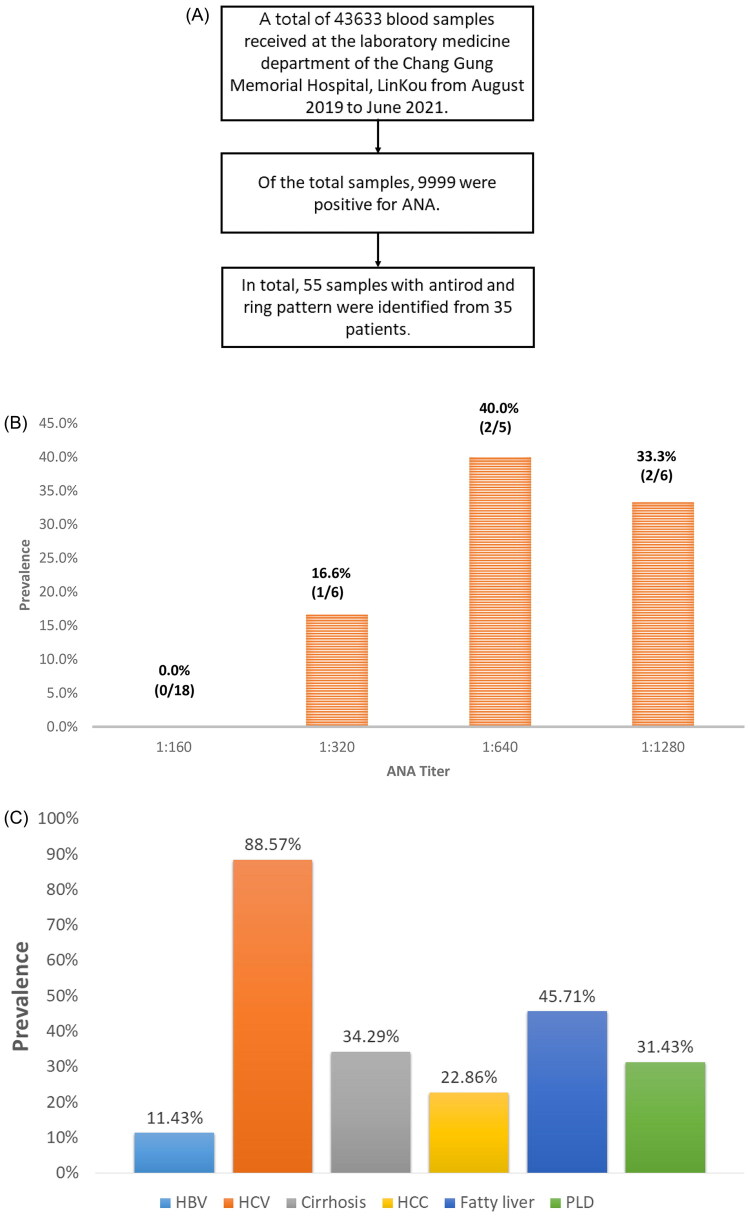
** **(A) Flowchart illustrating the selection of patients with the ‘rods and rings’ (RR, AC-23) antinuclear antibody pattern from the total cohort (2019–2021). (B) Correlation between cerebrovascular disease (CVD) and antinuclear antibody (ANA) titres among RR-positive patients. Patients with high ANA titres (≥1:640) showed a significantly higher frequency of CVD (*p* = .026). (C) Hepatic manifestations of 35 patients with the RR pattern, including proportions of fatty liver, liver cirrhosis, parenchymal liver disease and hepatocellular carcinoma (HCC). Abbreviations: ANA: antinuclear antibody; CVD: cerebrovascular disease; HBV: hepatitis B virus; HCV: hepatitis C virus; HCC: hepatocellular carcinoma; PLD: parenchymal liver disease; RR: rods and rings.

We initially collected 43,633 samples, 9999 (22.9%) of which were positive for ANA. Of the positive samples, only 55 samples (0.6%) from 35 patients (22 men, 13 women; mean age, 68 years) displayed the AC-23 pattern (Figure 1(A)). The demographic data, clinical characteristics and ANA features of these patients are presented in Table 1. The underlying autoimmune-related diseases included autoimmune diseases in six patients, including psoriasis in two patients, vitiligo in two patients, scleroderma in one patient and autoimmune hepatitis in one patients. Regarding ANA features, a titre of 1:160 was most frequent (51.4%). Thirty-one (88.6%) RR-positive patients had positive HCV serology and 12 (34.3%) patients developed liver cirrhosis. Eight patients had hepatocellular carcinoma (HCC), all of these patients were infected with HCV. The most frequent reason for ANA testing was hepatic problems (42.9%), and ANA tests were generally ordered by hepatologists to assess abnormal liver function.

**Table 1.** Demographic, clinical and laboratory characteristics of 35 patients exhibiting the ‘rods and rings’ (RR, AC-23) antinuclear antibody pattern.

Characteristics	*N* = 35 (%)
Demographic	
Age, years (mean ± standard deviation)	68.49 ± 9.39 (100)
Male sex	22 (62.9)
Clinical characteristics	
Diabetes	13 (37.1)
Hypertension	16 (45.7)
Hyperlipidaemia	10 (28.6)
Cerebrovascular disease	5 (14.3)
Pulmonary disease	3 (8.6)
Immune-related disease	6 (17.1)
Malignancy	13 (37.1)
Thyroid disease	2 (5.7)
Chronic kidney disease	7 (20)
Hepatitis B	4 (11.4)
Hepatitis C	31 (88.6)
Cirrhosis	12 (34.3)
Former alcohol consumption	8 (22.9)
Reason for the ANA test	
Neurologic problem	3 (8.6)
Ophthalmologic problem	2 (5.7)
Pulmonary problem	3 (8.6)
Hepatic problem	15 (42.9)
Renal problem	3 (8.6)
Dermatologic problem	6 (17.1)
Hematologic problem	2 (5.7)
Infectious problem	1 (2.9)
ANA titre	
1:160	18 (51.4)
1:320	6 (17.1)
1:640	5 (14.3)
1:1280	6 (17.1)

*Notes:* Cerebrovascular disease included stroke, coronary arterial disease, peripheral arterial occlusive disorder and atrial fibrillation. Data are presented as *n* (%) or mean ± standard deviation, unless otherwise specified. Statistical analyses were performed using the Mann–Whitney *U* test or Fisher’s exact test (two-tailed).

Abbreviations: ANA: antinuclear antibody; RR: rods and rings; CVD: cerebrovascular disease; HCV: hepatitis C virus; HCC: hepatocellular carcinoma.

Four patients (11.4%) presented with diseases unrelated to HCV infection, and only one of these patients had an autoimmune disease (Table 2). There were no specific characteristics or underlying diseases in these four patients. The drugs used by these patients in the prior six months before enrolment were mainly for chronic disease. Interestingly, three of four patients had used beta-blockers, including bisoprolol, propranolol and timolol eye drops. One patient died because of lung cancer, and others have undergone regular follow-up in our outpatient clinic.

**Table 2.** Demographic and clinical characteristics of patients with anti-RR antibodies without hepatitis C virus (HCV) infection.

Subjects	ANA titre	Sex	Age	Autoimmune diagnosis	Concomitant diseases	Reason for the ANA test	Other autoantibodies	Clinical outcome	Medication used six months before the ANA test
1	1:320	M	70	Scleroderma	DM, AF	Pneumonitis	Mi-2 alpha	Cardiology, neurology and rheumatology follow-up	Cortisone, glimepiride, bisoprolol, edoxaban, diphenidol
2	1:160	M	84	None	DM, CKD, HTN, hyperlipidaemia	Anaemia	Not performed	Nephrology follow-up	Spironolactone, folic acid, propranolol, tamsulosin, furosemide, antacid
3	1:320	M	62	None	CKD, HTN, hyperlipidaemia	Renal insufficiency	Not performed	Nephrology follow-up	Unknown HTN drug, latanoprost eye drops, Combigan eye drops (brimonidine tartrate 0.2%/timolol 0.5%)
4	1:640	F	60	None	Lung cancer	Dyspnoea	Not performed	Dead	None

Abbreviations: DM: diabetes mellitus; AF: atrial fibrillation; CKD: chronic kidney disease; HTN: hypertension.

### Differences between patients with high and low ANA titres

We also analyzed patient groups with high (≥1:640) and low (≤1:320) ANA titres (Table 3). Demographic data, clinical characteristics and laboratory data did not significantly differ between these groups, excluding the significantly higher incidence of cerebrovascular disease in the high ANA titre group (*p* = .026). Cerebrovascular disease was present in 0 of 18 patients with an ANA titre of 1:160, 1 of 6 patients with an ANA titre of 1:320, 2 of 5 patients with an ANA titre of 1:640 ANA and 2 of 6 patients with an ANA titre of 1:1280 (Figure 1(B)).

**Table 3.** Comparison of demographic, clinical and laboratory characteristics between patients with low (≤1:320) and high (≥1:640) ANA titres.

Characteristics	Low ANA titre (≤1:320) *N* = 24	High ANA titre (≥1:640) *N* = 11	*p*
Demographics			
Age, years	67.9 ± 9.2	68.5 ± 9.4	.494
Male sex	17	5	.258
Clinical characteristics			
Diabetes	9	4	.626
Hypertension	12	4	.452
Hyperlipidaemia	7	3	.685
Cerebrovascular disease	1	4	.026*
Pulmonary disease	2	1	.691
Immune -related disease	3	3	.352
Malignancy	8	5	.708
Thyroid disease	2	0	.468
Chronic kidney disease	6	1	.392
Hepatitis B	1	3	.082
Hepatitis C	21	10	.628
Liver cirrhosis	7	5	.451
Use of ribavirin for HCV infection (missing N)	16 (*N* = 3)	8 (*N* = 2)	.285
Laboratory finding (missing N)			
WBCs (*N* = 13)	7900 ± 3726	6600 ± 2724	.407
Hb (*N* = 12)	12.7 ± 2.8	11.9 ± 1.9	.319
Platelets, ×10^3^ (*N* = 13)	215.2 ± 90.2	162.7 ± 96.3	.329
Creatinine (*N* = 18)	0.98 ± 0.45	0.89 ± 0.67	.296
AST (*N* = 18)	63.9 ± 65.2	53.8 ± 36.5	1.000
ALT (*N* = 14)	52.5 ± 29.4	47.6 ± 22.3	.856

*Notes:* Data were compared by the Mann–Whitney U test or Fisher’s exact test. Data are presented as *N* (%) or the mean ± standard deviation. ‘Missing *N*’ denotes the number of patients for whom data were missing.

Abbreviations: ANA: antinuclear antibody; HCV: hepatitis C virus; WBCs: white blood cells; Hb: haemoglobin; AST: aspartate aminotransferase; ALT: alanine aminotransferase. *p < 0.05.

### Clinical differences in RR-positive patients according to the presence or absence of HCV infection

In total, 31 of 35 RR-positive patients had HCV infection (Table 4). The mean age of the HCV-positive patients was 68.4 ± 9.4 years, and the majority of these patients were males (61.3%). Hepatic sonography revealed HCC and liver cirrhosis in 8 (25.8%) and 12 (38.7%) of these patients, respectively. However, fatty liver (16/31, 51.6%) was the most common disease in HCV-positive patients. Despite some missing data, abnormal liver function was more common in HCV-positive patients than in HCV-negative patients (*p* < .05). HCV genotype 1 was most prevalent, being present in 17 HCV-positive patients (54.8%). In the analysis of previous treatments used for hepatitis C, five patients with no data were excluded. Of the remaining 26 patients, 24 (92.3%) were using RBV, mainly in combination with IFN or direct-acting antivirals (DAAs).

**Table 4.** Clinical and laboratory characteristics of RR-positive patients stratified by the presence or absence of hepatitis C virus (HCV) infection.

Characteristics	HCV (+) *N* = 31	HCV (−) *N* = 4	*p*
Demographics			
Age, years	68.4 ± 9.4	69 ± 10.9	.861
Males	19	3	.522
ANA titre			
1:160	17	1	NS
1:320	4	2	NS
1:640	4	1	NS
1:1280	6	0	NS
High ANA titre (≥1:640)	10	1	.628
Laboratory finding (missing N)			
WBCs (*N* = 13)	7705 ± 3672	6533 ± 1795	.718
Hb (*N* = 12)	12.7 ± 2.4	11.0 ± 4.4	.573
Platelets, ×10^3^ (*N* = 13)	189.7 ± 87.7	271.7 ± 111.5	.265
Creatinine (*N* = 18)	1.0 ± 0.5	0.8 ± 0.3	.785
AST (*N* = 18)	65.8 ± 59	19.5 ± 3.5	.015*
ALT (*N* = 14)	54.7 ± 25	14 ± 4.2	.037*
Hepatic sonography			
Hepatocellular carcinoma	8	0	.553
Liver cirrhosis	12	0	.275
Fatty liver	16	1	.608
Parenchymal liver disease	11	1	.575
Former alcohol consumption	8	0	.553
HCV characteristics			
Genotype 1	17		
Genotype 2	9		
Previous treatment for HCV (*N* = 26)			
Treatment with ribavirin (%)	24 (92.3)		
Unknown	5		

*Notes:* Data were compared by the Mann–Whitney *U* test or Fisher’s exact test. Data are presented as *N* (%) or the mean ± standard deviation. ‘Missing *N*’ denotes the number of patients for whom data were missing.

Abbreviations: RR: rods and rings; HCV: hepatitis C virus; ANA: antinuclear antibody; WBCs: white blood cells; Hb: haemoglobin; AST: aspartate aminotransferase; ALT: alanine aminotransferase. *denotes statistical significance (*p* < 0.05)

Meanwhile, 45.71% of patients presented with fatty liver (45.71%) and cirrhosis, parenchymal liver disease, and HCC were detected in 34.29%, 31.43% and 22.86% of patients, respectively (Figure 1(C)). Meanwhile, 11.43% of patients were also positive for hepatitis B.

Alcohol consumption can affect the liver manifestations of hepatitis C infection. Our article included eight patients (22.6%) who had alcohol consumption history and all had hepatitis C infection. Four of them developed HCCs, five had cirrhosis and two had high ANA titre (Supplementary Table S1).

## Discussion

From patients’ and rheumatologists’ viewpoints, ANA is mainly used to screen for autoimmune diseases. In this study, 9999 of 43,633 samples were positive for ANA (22.9%), but only 55 samples (0.56%) from 35 patients displayed presented the anti-RR ANA pattern. Shaikh et al. estimated that the prevalence of anti-RR autoantibodies in the general population in the National Health and Nutrition Examination Survey was 0.823% [23]. In the Chinese population, the frequency of anti-RR antibodies among ANA-positive patients was low (0.18–0.49%) [20,24].

Recently, the anti-RR pattern was reported to be highly associated with hepatitis C treatment [10,13–17]. In our study, 92.3% of patients with anti-RR antibodies were receiving RBV-based and treatment for hepatitis C. In previous studies, the frequency of anti-RR antibodies ranged from 53% to 96% [10,25]. RBV plays an important role in reducing the burden of HCV infection. The literature indicated that if only DAAs are used, anti-RR antibodies will not be produced [17]. RBV, a guanosine-like drug, inhibits IMPDH, resulting in the depletion of GTPs required for viral RNA synthesis [15]. RBV-induced anti-RR autoantibodies appear to be associated with a more frequent nonresponse to IFN/RBV therapy and a significantly higher HCV viral load [26].

In our study, four patients without a past medical history of hepatitis C or IFN/RBV treatment developed anti-RR antibodies. One of these patients had scleroderma, and another had lung cancer. In addition, three of four patients used multiple drugs. In particular, all three patients were on beta-blockers. We also noted the presence of alcohol use. Although all eight patients who had ever drunk alcohol had hepatitis C, four of them had HCC. This increased risk of HCC is likely due to the relationship between hepatitis C and alcohol consumption [27]. However, previous literature reviews did not find any association between alcohol and anti-RR antibody. In addition, there was no clear direct correlation between alcohol and IMPDH.

A review by Calise and Chan reported rare cases of anti-RR antibodies in diseases other than hepatitis C, including autoimmune diseases treated with mycophenolic acid (MPA) or methotrexate [13]. Anti-RR formation can be induced *in vitro* by IMPDH inhibitors [13]. However, beta-blockers do not inhibit this enzyme. Shaikh et al. found that 38 of 39 hepatitis C-naïve patients with anti-RR autoantibodies received poly-pharmacy [23]. Climent et al. reported the presence of anti-RR antibodies in 14 patients with autoimmune diseases [28]. In a Japanese study, the authors also mentioned that patients with pancreatitis also developed anti-RR antibodies [29]. This RR structure is found in the pancreas and spleen. Upon damage to these organs, proteins become exposed, which could induce anti-RR production.

Another finding of this study was that patients with high ANA titres were more likely to have **cerebro**vascular disease than those with low ANA titres. Several risk factors for **cerebro**vascular disease, including inflammation, have been described. The incidence of traditional risk factors, including male sex, age, diabetes, hypertension and hyperlipidaemia, did not differ between patients with low and high ANA titres in this study. In a retrospective study in Southwest China, Zhang et al. found that the serum lipid, glucose and uric acid levels were significantly higher in patients with anti-RR antibodies than in healthy controls [24]. It was also assumed that anti-RR antibodies might be related to metabolic disorders in patients without hepatitis. Generally, patients with higher ANA titres have a higher risk of inflammation than patients with lower ANA titres. Selmi et al. reported that ANA positivity increased the risk of connective tissue disease over 15 years in the general population, and the highest risk was noted for ANA titres ≥ 1:160 (hazard ratio = 14.19, 95% confidence interval = 3.07–65.68) [30]. In addition, many inflammation-relevant diseases have been specially characterized by high IMPDH expression in rapidly proliferating immunocytes. IMPDH is an attractive target for immunosuppressive agents, especially MPA [13], and anti-RR antibody targets an antigen related to IMPDH. A high ANA titre (anti-RR pattern) might lead to stronger inflammatory responses, suggesting the presence of inflammatory diseases, including cerebrovascular disease. However, because of the small number of cases, further research is required.

Although Fisher’s exact test did not reveal statistically significant differences (*p* < .05), patients with former alcohol consumption exhibited higher rates of liver cirrhosis (62.5%) and hepatocellular carcinoma (50%) compared with non-drinkers (25.9% and 14.8%, respectively). This trend supports a possible synergistic or additive effect of alcohol exposure on hepatic injury, consistent with previous epidemiologic data suggesting that alcohol may accelerate liver disease progression but is not directly associated with ANA titres (Supplementary Table S1).

## Conclusion

Beyond its association with hepatitis C treatment, the presence of anti-RR antibodies might also indicate a risk for cerebrovascular disease, particularly at high titres. This finding deserves the attention of clinicians and highlights new avenues of research.

## Supplementary Material

Supplementary Table S1_AC23_AoM_TMC1019V3.docx

## Data Availability

The datasets generated and/or analyzed during the current study are not publicly available due to institutional and legal restrictions governed by the Chang Gung Memorial Hospital Institutional Review Board and the Personal Data Protection Act in Taiwan. De-identified data supporting the findings of this study are available from the corresponding author (Tien-Ming Chan) **upon reasonable request**, subject to approval by the Institutional Review Board and execution of a data use agreement. The analysis code and detailed variable definitions will also be shared **upon reasonable request**.
