# Supplementary material for: Unmasking the ‘rods and rings’ antinuclear antibody pattern: a sign of hepatitis C treatment and a risk factor for cerebrovascular disease
Source: Ann Med. 2025 Dec 18;57(1):2600753. doi: 10.1080/07853890.2025.2600753 (PMC12720668; doi:10.1080/07853890.2025.2600753)
Supplement: Supplementary Table S1_AC23_AoM_TMC1019V3.docx [file IANN_A_2600753_SM2117.docx]

**Supplementary Table S1.** Comparison of clinical characteristics between RR-positive patients with and without former alcohol consumption

| Variable | Former alcohol consumption (n = 8) | Never alcohol consumption (n = 27) | p-value |
| --- | --- | --- | --- |
| Male | 7 (87.5%) | 15 (55.6%) | 0.21 |
| Liver cirrhosis | 5 (62.5%) | 7 (25.9%) | 0.09 |
| HCC | 4 (50.0%) | 4 (14.8%) | 0.07 |
| High ANA titer | 2 (25.0%) | 9 (33.3%) | 0.69 |

Abbreviations: RR, rods and rings; HCC, hepatocellular carcinoma; ANA, antinuclear antibody.
